# Ligand-specific duality of aryl hydrocarbon receptor signaling in cognitive health: from environmental neurotoxicity to microbiome-mediated neuroprotection

**DOI:** 10.3389/fnins.2026.1823961

**Published:** 2026-06-24

**Authors:** Chong Tian, Shan Yang, Xinyao Zhang, Hongyu Yan

**Affiliations:** 1 School of Nursing, Tongji Medical College, Huazhong University of Science and Technology, Wuhan, China; 2 Department of Nursing, Union Hospital of Tongji Medical College, Huazhong University of Science and Technology, Wuhan, China; 3 Department of Geriatrics, Union Hospital, Tongji Medical College, Huazhong University of Science and Technology, Wuhan, China

**Keywords:** aryl hydrocarbon receptor, cognitive function, ligand-specific signaling, neuroprotection, neurotoxicity

## Abstract

The aromatic hydrocarbon receptor (AhR) is a key molecular interface integrating environmental chemical signals with host-microbiome metabolism, with profound effects on brain function. This review systematically addresses the ligand-specific duality of AhR signaling in cognitive health, comparing the predominantly neurotoxic signaling driven by environmental polycyclic aromatic hydrocarbons (PAHs) with the predominantly neuroprotective signaling mediated by gut microbiota-derived tryptophan metabolites. However, this dichotomy is context-dependent rather than absolute. PAHs activate AhR in a sustained, high-affinity manner, engaging downstream NF-κB neuroinflammation, NLRP3 inflammasome activation, oxidative stress, synaptic dysfunction, and transgenerational epigenetic alterations. In contrast, microbiota-derived metabolites such as indole-3-propionic acid (IPA) and kynurenic acid (KYNA) elicit transient, low-affinity AhR activation that engages cell-type-specific programs promoting anti-inflammatory responses, neurogenesis, blood–brain barrier integrity, and neuronal homeostasis. Critically, the outcome of AhR activation is modulated by ligand pharmacokinetics, cell-type identity, temporal dynamics of receptor engagement, and tissue-specific co-factor availability. These contextual variables determine whether AhR functions as a driver of neurodegeneration or a guardian of cognitive resilience. We further examine the divergent roles of AhR in Alzheimer’s and Parkinson’s diseases, where the balance between detrimental and protective ligands determines disease progression. Finally, we discuss therapeutic strategies targeting the AhR–gut–brain axis, including dietary modulation, probiotic interventions, and selective AhR modulators. Understanding the context-dependent outcomes of AhR activation provides a framework for developing precision approaches to preserve cognitive function and prevent neurodegeneration.

## 1 Introduction

The relationship that connects environmental exposure, host metabolism, and brain health is intricate and has become a central focus of modern neuroscience. At the crossroads of these interactions lies the aryl hydrocarbon receptor (AhR), a ligand-activated transcription factor historically recognized for its role in mediating the toxicity of environmental pollutants such as dioxins (Stockinger et al., 2014). Beyond its xenobiotic function, AhR is now understood as a critical sensor and integrator of a wide array of endogenous and microbiome-derived signals, profoundly influencing immune regulation, cellular homeostasis, and nervous system function (Neavin et al., 2018; Sondermann et al., 2023). This dual nature positions AhR as a molecular switch capable of driving divergent physiological outcomes. Its activation by structurally diverse ligands can lead to either protective or deleterious effects, a paradox particularly evident in the context of cognitive function and neurodegenerative diseases.

Two major classes of endogenous AhR ligands, which are polycyclic aromatic hydrocarbons (PAHs) and metabolites of the essential amino acid tryptophan, have emerged as key modulators in this paradigm. Polycyclic aromatic hydrocarbons (PAHs) are ubiquitous environmental pollutants that are generated by the incomplete combustion of organic materials and that exist widely in urban air, soil, and certain foods (Chepelev et al., 2015; Crépeaux et al., 2012). Exposure to polycyclic aromatic hydrocarbons, especially during critical developmental windows, is epidemiologically and experimentally associated with cognitive deficits, impaired neurodevelopment, and increased risk of neurobehavioral disorders (Chen et al., 2012; Perera et al., 2009, 2014). In stark contrast, a range of tryptophan metabolites, particularly those produced by commensal gut microbiota, such as indole-3-propionic acid (IPA) and kynurenoic acid (KYNA), have received attention for their neuroprotective properties (Owe-Larsson et al., 2025; Serger et al., 2022; Yin et al., 2023). These metabolites are the product of a complex interaction between host physiology and the gut microbiota, forming a central component of the microbiota-gut-brain axis (Gilbert et al., 2018; Nicholson et al., 2012).

Yet this specificity does not operate in isolation. The ultimate cognitive consequence of AhR activation is shaped by an integrated set of contextual variables: ligand binding affinity and pharmacokinetics, cell-type identity and its epigenetic landscape, the temporal dynamics of receptor engagement, and tissue-specific co-factor availability. Within this multi-dimensional framework, environmental PAHs predominantly direct AhR toward neurotoxic outcomes, while gut microbiota-derived tryptophan metabolites tend to engage AhR in a neuroprotective mode. However, exceptions exist in both directions, as discussed throughout this review.

## 2 Aryl hydrocarbon receptor: a janus-faced regulator at the nexus of environment and metabolism

The aryl hydrocarbon receptor is a phylogenetically conserved, ligand-dependent basic helix-loop-helix/Per-Arnt-Sim (bHLH/PAS) transcription factor. In its inactive state, AhR resides in the cytoplasm as part of a multiprotein complex that includes chaperones like HSP90 and the co-chaperone p23 (Farooqi et al., 2023). Upon binding to an agonist ligand, AhR undergoes a conformational change, translocates to the nucleus, sheds its chaperone complex, and heterodimerizes with its partner, the AhR nuclear translocator (ARNT). This active complex binds to specific DNA sequences known as xenobiotic response elements (XREs) or dioxin response elements (DREs) in the promoter regions of target genes, initiating their transcription (Sondermann et al., 2023; Stockinger et al., 2014). The classic and most studied AhR target gene is *CYP1A1*, which encodes a cytochrome P450 enzyme central to the phase I metabolism of xenobiotics. However, AhR regulates a vast and diverse transcriptome, influencing pathways involved in cell cycle regulation, immune response, differentiation, and oxidative stress management (Farooqi et al., 2023; Grishanova and Perepechaeva, 2022).

This functional duality arises from at least four deeply intertwined contextual variables: ligand binding affinity and pharmacokinetics; cell-type identity and its epigenetic landscape; the temporal dynamics of AhR activation; and tissue-specific co-factors and chromatin accessibility. These dimensions interact to generate a highly plastic signaling output that the simplified PAH-versus-tryptophan binary cannot fully capture, and they must be held in view throughout the mechanistic analysis that follows. AhR ligands span a wide structural spectrum, from planar hydrophobic environmental toxicants to smaller, more hydrophilic endogenous and dietary molecules (Gutiérrez-Vázquez and Quintana, 2018; Huang et al., 2023). High-affinity exogenous ligands—such as TCDD and BaP—produce sustained, potent AhR activation typically associated with toxicity and dysregulation (Chepelev et al., 2015; Murray et al., 2014), whereas low-affinity endogenous ligands such as tryptophan metabolites tend to elicit transient or context-dependent activation (Dong et al., 2020; Ma et al., 2020). These differences in binding affinity and pharmacokinetics ultimately determine the duration, intensity, and transcriptional profile of the response (Sondermann et al., 2023).

This divergence in signaling is driven not only by ligand affinity and activation kinetics, but also by distinct transcriptional and cell-specific immune programs. High-affinity environmental ligands such as benzo[a]pyrene (BaP) promote prolonged AhR/ARNT nuclear retention, sustained CYP1A1/CYP1B1 induction, and altered NMDA receptor subunit expression, thereby disrupting glutamatergic signaling and synaptic plasticity (Chepelev et al., 2015). In contrast, microbiota-derived tryptophan metabolites such as indole and indole-3-propionic acid (IPA) induce more transient AhR activation and preferentially suppress NF-κB-dependent inflammatory transcription while enhancing neuroprotective pathways (Owe-Larsson et al., 2025; Rothhammer et al., 2016). These ligand-dependent effects are further shaped by cellular context. In astrocytes, microbial AhR ligands induce SOCS2-dependent suppression of Ccl2, Csf2, and Nos2 expression, limiting CNS inflammation (Rothhammer et al., 2016). In dendritic cells and T cells, AhR activation interacts with the IDO1-kynurenine pathway to regulate the balance between FoxP3^+^ regulatory T cells and pro-inflammatory TH17 responses in a ligand-dependent manner (Mezrich et al., 2010; Quintana et al., 2008). In addition, microbial metabolites such as butyrate can epigenetically potentiate AhR-mediated anti-inflammatory transcription through histone deacetylase inhibition (Modoux et al., 2022). Together, these findings indicate that AhR functions as a context-dependent signaling hub. Its downstream effects are determined by ligand identity, transcriptional dynamics, and cell-specific regulatory networks.

Cellular context is equally critical. AhR is expressed in a wide variety of cell types within the nervous system and the periphery, including neurons, astrocytes, microglia, endothelial cells, and numerous immune cells (Huang et al., 2023; Perepechaeva and Grishanova, 2020). The outcome of AhR activation is determined by the composition of cell-specific co-activators, co-suppressors and other transcription factors. Notably, AhR acts differently in tumor-associated macrophages in glioblastoma, where its activation creates an immunosuppressive environment that supports cancer progression (Campesato et al., 2020; Takenaka et al., 2019). This context is distinct from the neuroprotective roles described above.

**FIGURE 1:**
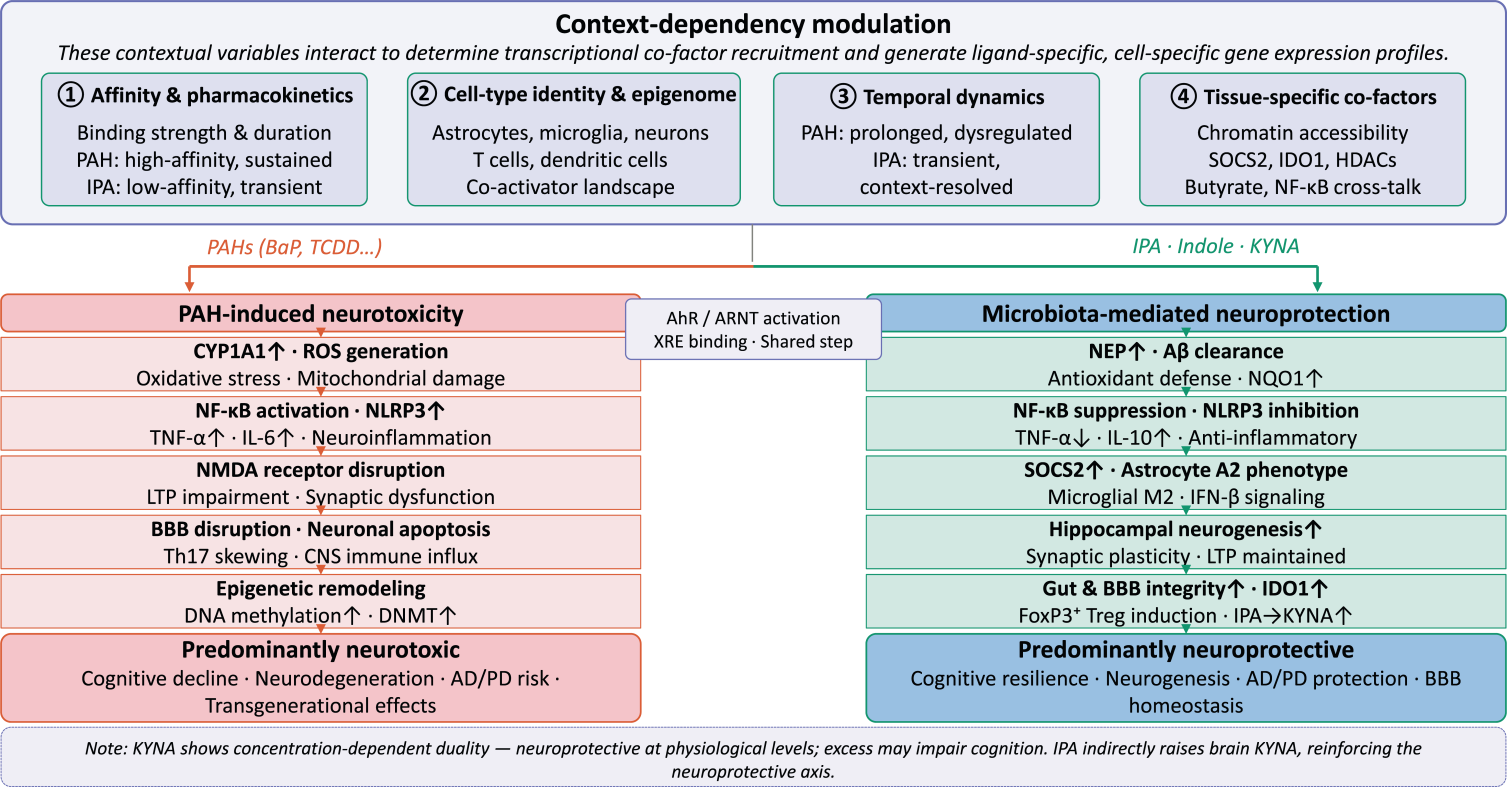
Ligand-specific divergent signaling of aryl hydrocarbon receptor (AhR) in the brain.

Furthermore, AhR signaling extends beyond the canonical AhR/ARNT/XRE pathway. Non-canonical mechanisms involve cross-talk with other major signaling cascades. AhR can interact with transcription factors including NF-κB, estrogen receptors, and hypoxia-inducible factors, modulating their activity independently of ARNT and XRE binding (Sondermann et al., 2023). AhR also acts as an E3 ubiquitin ligase. It targets certain proteins like the estrogen receptor for proteasomal degradation (Farooqi et al., 2023). AhR has two opposing roles under oxidative stress. It increases expression of antioxidant enzymes such as NQO1. It also raises reactive oxygen species by inducing CYP enzymes. It thus acts as a “double agent” in redox balance (Grishanova and Perepechaeva, 2022). AhR has complex regulatory functions as a master integrator of environmental and metabolic signals. AhR links external chemical exposures to internal metabolism. External exposures include pollutants such as PAHs. Internal metabolism is shaped by diet and the gut microbiome (De Vos et al., 2022; Ma et al., 2020). Therefore, the final output of this receptor to processes such as neuroinflammation, neuron survival, and synaptic function, and ultimately to cognitive health, is the product of specific ligand-receptor-cell axis interactions and is not predetermined. Understanding this flexibility is critical. It helps explain why AhR has opposing roles in neurotoxicity and neuroprotection (Figure 1 and Supplementary Figure 1).

## 3 The neurotoxic axis—PAH-AhR signaling and its detrimental impact on cognitive function

Polycyclic aromatic hydrocarbons are major environmental toxicants which can activate AhR. Their neurotoxic effects are well documented. Epidemiological studies provide compelling evidence linking prenatal and childhood PAH exposure to adverse cognitive outcomes. Studies in urban populations show clear effects. Higher prenatal PAH exposure links to lower IQ in 5-year-olds (Perera et al., 2009). It also links to developmental delay at age 3 (Perera et al., 2006) and more ADHD symptoms (Perera et al., 2014). These associations persist after adjustment for multiple potential confounders, suggesting a direct toxic effect on the developing brain.

Experimental models, especially those using zebrafish and rodents, have played an important role in elucidating these mechanisms. Early exposure to PAHs such as BaP causes long-lasting neurobehavioral changes, including hyperactivity in larvae and, in adulthood, lasting deficits in learning, memory, and anxiety-related behaviors (Chen et al., 2012; Knecht et al., 2017a,b). These effects are observed at exposure levels where PAHs are developmentally toxic but not overtly teratogenic, indicating a specific vulnerability of the nervous system (Billiard et al., 2008). Crucially, studies using zebrafish with the AHR2 gene knocked out have shown that the juvenile hyperkinetic phenotype induced by BaP is partially dependent on the zebrafish homologous gene AHR2. This finding confirms the receptor’s role in mediating certain neurobehavioral effects (Knecht et al., 2017b).

Benzo[a]pyrene causes neurotoxicity through a series of events. These events are sequentially mediated by AhR (Chepelev et al., 2015). Initial binding and activation of AhR leads to the modulation of gene expression, including subunits of the N-methyl-D-aspartate (NMDA) glutamate receptor. Changes in NMDA receptor function disrupt neuronal activity and impair long-term potentiation, which underpins learning and memory, ultimately leading to cognitive decline. This pathway provides a plausible link between molecular initiating events (AhR activation) and the observed apical effect (learning and memory deficits). PAH mixtures can cause developmental and neurotoxicity. Their effects are not simply additive. In real-world environments, organisms are invariably exposed to complex PAH mixtures rather than individual compounds. A representative Superfund mixture (SM10), constructed from the 10 most abundant PAHs at the Portland Harbor Superfund site, comprised pyrene, fluoranthene, retene, benzo[a]anthracene, chrysene, naphthalene, acenaphthene, phenanthrene, fluorene, and 2-methylnaphthalene. These compounds accounted for 76%–87% of total PAH mass at the site (Geier et al., 2018). These constituents differ markedly in AhR binding affinity, CYP1A-inducing potency, and metabolic lability. Notably, fluoranthene inhibits CYP1A activity in PAH mixtures and can potentiate the embryotoxic effects of AhR agonists such as benzo[a]anthracene (Billiard et al., 2008). Developmental exposure to SM10 in zebrafish induced a broader range of morphological deficits than any single constituent and caused persistent neurobehavioral impairments in adults, including impaired learning and reduced habituation to startle stimuli (Geier et al., 2018). Such non-additive effects likely reflect the involvement of multiple pathways, including AhR signaling, CYP1-mediated formation of reactive metabolites, and oxidative stress, making the toxicity of PAH mixtures difficult to predict from single compounds alone (Billiard et al., 2008; Geier et al., 2018). These mixture effects complicate risk assessment but underscore the real-world relevance of PAH exposure.

Polycyclic aromatic hydrocarbon-AhR signaling affects more than neurons. It also targets non-neuronal cells and systemic pathways. This further disrupts cognitive function. Perinatal PAH exposure in rats increases anxiety-like behavior in adulthood. It also reduces neuronal metabolism in the hippocampus and amygdala (Crépeaux et al., 2012). This finding indicates an important effect. PAHs cause long-term changes in brain energy metabolism and emotional regulation circuits. Furthermore, PAH exposure can induce systemic and neuroinflammation, a known driver of cognitive decline. Although not all PAHs are strong AhR agonists, many can induce cytochrome P450 enzymes via AhR, leading to the generation of reactive oxygen species and oxidative stress, which damage neurons and glial cells (Goodale et al., 2013; Grishanova and Perepechaeva, 2022).

The impact of PAHs is not limited to the individual exposed; transgenerational effects have been observed. Developmental exposure to BaP causes neurobehavioral and physiological deficits in zebrafish. These include social anxiety-like behavior and altered metabolic profiles. These deficits can be passed to unexposed offspring (F2) raised in clean water (Knecht et al., 2017a). These transgenerational effects link to altered DNA methylation and DNMT expression. They depend on AHR2-mediated epigenetic changes. This finding broadens our understanding of PAH neurotoxicity. Early damage can program long-lasting cognitive and behavioral risks across generations via epigenetic remodeling.

In summary, PAH-AhR signaling harms cognition through several key pathways. It disrupts synaptic plasticity and glutamate signaling. It induces oxidative stress and neuroinflammation. It changes brain metabolism. It may also cause long-lasting and transgenerational epigenetic changes. This axis represents a significant pathway through which environmental pollution can compromise brain function and contribute to the risk of neurodevelopmental and cognitive disorders.

## 4 The neuroprotective axis—gut microbiota-derived tryptophan metabolites and AhR-mediated homeostasis

In contrast to the neurotoxic PAH axis, AhR activation by gut microbiota-derived tryptophan metabolites is strongly linked to neuroprotection and homeostasis. Tryptophan catabolism proceeds through three routes with distinct cellular origins (Agus et al., 2018; Roth et al., 2021; Gao et al., 2020). The serotonin/melatonin pathway is mediated by host enzymes, mainly TPH1 in enterochromaffin cells and TPH2 in neurons, and its metabolites are not considered direct AhR agonists. The kynurenine pathway is predominantly host-driven via IDO1/IDO2 and TDO, though the gut microbiota shapes its flux indirectly through TLR-mediated IDO induction and butyrate-dependent IDO suppression (Gao et al., 2020); KYNA and kynurenine can cross the blood–brain barrier and act as AhR ligands. The indole pathway is microbiota-dependent. Commensal bacteria such as *Clostridium sporogenes*, *Lactobacillus*, and *Bacteroides* convert tryptophan into indole derivatives including IPA, IAA, ILA, and IAld, many of which function as gut-derived AhR agonists with neuroprotective effects (Agus et al., 2018; Gao et al., 2020).

Indole-3-propionic acid (IPA) is a key example. It is a microbiota-derived AhR ligand with neuroprotective effects. Produced by specific gut bacteria such as *C. sporogenes*, IPA exhibits multiple properties beneficial to the nervous system. IPA effectively scavenges free radicals. It lowers pro-inflammatory cytokines such as TNF-α. It also inhibits the NLRP3 inflammasome (Owe-Larsson et al., 2025). In experimental models, IPA has demonstrated remarkable efficacy. IPA improves axonal regeneration and recovery after nerve injury in mice. This effect requires the gut microbiome. It acts through an immune mechanism involving neutrophils (Serger et al., 2022). In models of aging and neurodegeneration, IPA and other indoles (e.g., indole-3-acetic acid, IAA) alleviate oxidative stress, inflammation, and neuronal apoptosis by activating the GPR30/AMPK/SIRT1 pathway (Yin et al., 2023). A synbiotic therapy was designed to promote IPA production. It used *C. sporogenes* and its preferred prebiotic xylan. In a transgenic Alzheimer’s disease mouse model, this therapy significantly improved cognition, reduced amyloid-β pathology, suppressed neuroinflammation, and restored synaptic ultrastructure (Li et al., 2024). Correlation analyses showed a clear relationship. Higher gut-derived IPA levels directly linked to better behavioral outcomes.

The kynurenine pathway, while also producing neurotoxic metabolites like quinolinic acid, generates AhR ligands with neuroprotective potential, most notably kynurenic acid (KYNA). KYNA acts as an antagonist of glutamate receptors and is generally considered neuroprotective, although this generalization requires immediate qualification. KYNA’s role is complex and concentration-dependent (Ostapiuk and Urbanska, 2022; Schwarcz et al., 2012). At physiological concentrations (low nanomolar), KYNA may preferentially inhibit α7 nicotinic receptors, potentially impairing cognitive function—a finding inconsistent with simple “neuroprotection.” At higher concentrations, NMDA receptor antagonism may indeed protect against excitotoxicity, but simultaneously impair synaptic plasticity necessary for learning. The therapeutic window for KYNA-mediated protection may be vanishingly narrow. The balance between neurotoxic and neuroprotective KP metabolites is crucial, and dysregulation toward the neurotoxic branch is implicated in several neurodegenerative and psychiatric disorders (Li et al., 2022; Savitz, 2020; Tanaka et al., 2020). Of note, KYNA is generated both endogenously by host cells and, to a lesser extent, by intestinal microbes, while IPA is exclusively produced by gut microbiota (Owe-Larsson et al., 2025). Intriguingly, the gut-derived IPA, which readily crosses the blood-brain barrier via passive diffusion and is not synthesized *in situ* within the brain, has been shown to directly elevate brain KYNA levels in rodents following oral administration, with subchronic feeding producing several-fold increases in both plasma and prefrontal cortical KYNA concentrations (Owe-Larsson et al., 2025). This stands in contrast to KYNA itself, which is largely excluded from the central compartment under physiological conditions despite peripheral synthesis, making gut-derived IPA an indirect but physiologically meaningful route to augment central neuroprotective kynurenergic tone (Ostapiuk and Urbanska, 2022). Within the neuroprotective axis, KYNA shows both beneficial and detrimental effects. Although it can limit excitotoxicity, excessive KYNA may also impair cognition and synaptic function. Its overall impact therefore appears to depend on concentration and disease context. Consequently, microbiota-derived IPA can influence the central KP by boosting brain levels of KYNA, thereby shifting the balance toward neuroprotection (Owe-Larsson et al., 2025).

This cross-pathway modulation represents an important mechanism, but the extent of IPA’s influence on central KP metabolism in humans remains unknown. Peripheral administration of IPA may not significantly alter brain kynurenine metabolism if BBB transport is limited or if local synthesis dominates. AhR activation by these tryptophan metabolites mediates neuroprotection through several cellular targets within the central nervous system. Astrocytes emerge as key effectors. We note, however, that the evidence for astrocyte-specific neuroprotection derives largely from experimental autoimmune encephalomyelitis (EAE) models. Whether these findings extend to other neurodegenerative conditions with distinct pathophysiologies—such as Alzheimer’s disease, characterized by protein aggregation rather than autoimmune demyelination—requires validation. In EAE, IFN-β signaling in astrocytes requires AhR to exert its anti-inflammatory effects (Rothhammer et al., 2016). Similarly, the drug laquinimod exerts beneficial effects partly through AhR activation in astrocytes (Kaye et al., 2016; Rothhammer et al., 2021). AhR activation in astrocytes can also be driven by microbial metabolites, which in turn modulate microglial control over astrocytes, suppressing CNS inflammation (Rothhammer et al., 2018). This glial-centric mechanism highlights how gut-derived signals can fine-tune the brain’s immune environment.

While glial cells are central effectors, AhR also coordinates neuroinflammatory tone through peripheral immune cells, whose signals ultimately reach the brain. In dendritic cells, AhR activation induces IDO1 and promotes tolerogenic antigen presentation—marked by increased IL-10 and reduced IL-12/IL-23—favoring FoxP3^+^ Treg differentiation over Th17 responses. This Treg/Th17 balance is ligand-dependent: microbiota-derived indoles and kynurenine promote immune tolerance, whereas persistent PAH exposure skews CD4^+^ T cells toward Th17 polarization, amplifying CNS inflammation (Mezrich et al., 2010; Quintana et al., 2008). AhR similarly reprograms myeloid cells, with tryptophan metabolites favoring anti-inflammatory macrophage phenotypes and pollutant-derived ligands driving the opposite. Collectively, these peripheral immune effects converge on the CNS by maintaining intestinal barrier integrity, limiting microbial product translocation, and restricting pathogenic immune cell infiltration across the blood–brain barrier. Nevertheless, translating these peripheral immune mechanisms into therapy remains challenging, as illustrated by laquinimod’s Phase III setbacks due to modest efficacy and cardiovascular adverse effects.

Aryl hydrocarbon receptor signaling in neural progenitor cells is another vital neuroprotective mechanism. Tryptophan-metabolizing gut microbes regulate adult hippocampal neurogenesis via AhR. The metabolite indole, produced by bacterial tryptophanase, elicits neurogenic effects in the adult mouse hippocampus, promoting the differentiation and maturation of new neurons (Wei et al., 2021). This effect is absent in AhR knockout mice, confirming the receptor’s essential role. Enhanced neurogenesis is a fundamental process for learning, memory, and cognitive flexibility, linking microbial metabolite signaling directly to the structural plasticity of the brain.

Aryl hydrocarbon receptor activation by tryptophan metabolites serves an important role. It helps maintain the integrity of the gut-brain axis. A damaged intestinal barrier allows pro-inflammatory molecules to enter the bloodstream. These molecules may then cross the blood-brain barrier. Metabolites such as IPA preserve gut barrier function. They reduce inflammation in the gut. They break the cycle of gut inflammation leading to brain dysfunction (Owe-Larsson et al., 2025; Salminen, 2023). The differential capacity of tryptophan catabolites to access the central compartment is a critical determinant of their neuroactive effects. Kynurenine readily crosses the BBB via large neutral amino acid transporters and serves as a major substrate for KYNA synthesis in astrocytes. In contrast, indole and tryptamine, unlike serotonin, are sufficiently lipophilic to enter the brain and activate central AhR signaling. Indeed, studies demonstrated that systemic administration of indole rescued adult hippocampal neurogenesis in germ-free mice in an AhR-dependent manner. This confirms that BBB-permeant microbial metabolites can directly modulate central AhR signaling (Wei et al., 2021). Indoxyl sulfate, by contrast, is actively transported across the BBB via the organic anion transporter OAT3 expressed in cerebral microvessels, and its pathological accumulation in chronic kidney disease activates vascular AhR in a manner that disrupts rather than preserves barrier integrity (Salminen, 2023). This shows a key principle. The source, context, and type of metabolite determine AhR’s effect on barrier homeostasis.

The neuroprotective axis relies on a symbiotic loop. A healthy gut microbiome converts dietary tryptophan into beneficial AhR agonists. Examples include IPA, indole, and KYNA. These ligands activate AhR in target cells, including astrocytes, neural progenitors, and immune cells. This activation drives transcriptional programs with multiple effects: suppressing inflammation, combating oxidative stress, supporting neurogenesis, and maintaining barrier integrity. This axis is a cornerstone of the microbiota-gut-brain dialogue, essential for cognitive resilience and a promising target for interventions aimed at preserving brain health.

## 5 Comparative analysis of PAH vs. tryptophan metabolite signaling in cognitive and neurodegenerative disorders

Aryl hydrocarbon receptor signaling shows clear ligand-specific duality. This is very obvious in major cognitive and neurodegenerative disorders. The same receptor can be co-opted into pathogenic cascades by environmental toxins. It can also be harnessed into protective pathways by host-microbiome metabolites. These divergent mechanisms lead to opposite clinical outcomes. This comparative analysis focuses on Alzheimer’s disease (AD) and Parkinson’s disease (PD) as prime examples.

In Alzheimer’s disease, the kynurenine pathway and AhR are deeply implicated, but their roles are nuanced and ligand-dependent (Cortés Malagón et al., 2024; Liang et al., 2022). The kynurenine pathway can shift toward neurotoxic metabolites. Quinolinic acid increases and kynurenic acid decreases. This shift links to neuroinflammation, excitotoxicity, and disease progression (Kupjetz et al., 2025; Tanaka et al., 2020). These KP metabolites can activate AhR. However, the effect of AhR activation in AD models is not uniformly detrimental. Studies show that activating AhR with certain ligands, such as the flavonoid diosmin or the endogenous ligand L-kynurenine, can upregulate the expression of neprilysin (NEP), a major endogenous enzyme that degrades amyloid-β (Aβ) (Qian et al., 2021). AhR-mediated NEP upregulation enhances Aβ clearance and reduces cognitive deficits in transgenic AD mice. This positions AhR as a promising therapeutic target for enhancing protein catabolism (Qian et al., 2021). Conversely, the tryptophan metabolite indoxyl sulfate accumulates in conditions such as chronic kidney disease. This metabolite is an AhR agonist and may promote AD pathogenesis by disrupting BBB integrity and vascular function (Salminen, 2023). This highlights an important point. Not all tryptophan metabolites are beneficial. Disease states such as kidney failure can turn a metabolite into a neurotoxic AhR ligand. Thus, AhR function in Alzheimer’s disease is balanced. Activation by beneficial ligands supports Aβ clearance and protection. Activation by harmful ligands worsens cerebrovascular dysfunction and neuroinflammation.

Parkinson’s disease has three key pathological features. These are α-synuclein aggregation, mitochondrial dysfunction, and neuroinflammation (Simon et al., 2020; Jurcau et al., 2023; Subramaniam and Chesselet, 2013). The gut-brain axis is strongly involved. Evidence suggests pathology may start in the gut. It can spread to the brain through the vagus nerve (Claudino Dos Santos et al., 2023; Hirayama and Ohno, 2021; Braak et al., 2003). Gut dysbiosis is a consistent feature in Parkinson’s disease. It is often characterized by reduced short-chain fatty acid producers and increased mucin-degrading bacteria, which may lead to greater intestinal permeability (Baizabal-Carvallo and Alonso-Juarez, 2020; Hirayama and Ohno, 2021; Shen et al., 2021). However, dysbiosis in PD may be secondary to disease-related changes in diet, medication use (particularly levodopa, which can be metabolized by gut bacteria), or autonomic dysfunction affecting gut motility. Establishing causality—that altered microbiota drive PD pathogenesis rather than result from it—requires longitudinal studies that are currently lacking. A disturbed microbiota-tryptophan-KYN-AhR triad is proposed as a component of PD pathogenesis (Iwaniak et al., 2024). We note that this “triad” model is largely theoretical, built on associative studies. The resultant imbalance in tryptophan metabolites could influence AhR signaling. On one hand, a deficiency in protective AhR ligands like IPA could reduce anti-inflammatory tone. On the other hand, increased production of other AhR-active metabolites might drive maladaptive immune responses. Furthermore, environmental PAH exposure is a recognized risk factor, and its neurotoxic AhR-mediated mechanisms directly overlap with key pathological processes in PD (Jurcau et al., 2023; Riederer et al., 2023). Thus, in PD, AhR may be activated by a combination of detrimental environmental ligands and a dysregulated profile of endogenous tryptophan metabolites. However, the relative contribution of each remains unknown. Does PAH exposure accelerate PD in individuals with pre-existing microbiome dysfunction? Or does microbiome depletion unmask the toxicity of ambient PAH exposure? These interactions have not been systematically investigated.

The contrast is also evident in neurodevelopmental and neuropsychiatric conditions. Prenatal PAH exposure is linked to lower IQ, ADHD symptoms, and interacts with maternal stress to worsen behavioral outcomes in children (Perera et al., 2009, 2013, 2014). This suggests that PAH-AhR signaling can predispose the developing brain to cognitive and behavioral vulnerabilities. In contrast, disruptions in tryptophan metabolism, often mediated by inflammation and potentially linked to gut dysbiosis, are heavily implicated in depression and other mood disorders (Li et al., 2022; Lukić et al., 2022). Here, the problem may be a relative deficit of beneficial, AhR-activating metabolites like those from the indole pathway, or an overabundance of neurotoxic KP metabolites, leading to imbalances in serotonin and glutamate systems. Interventions that restore a healthy microbiota and normalize tryptophan metabolite production, thereby modulating AhR signaling, show promise for improving depressive symptoms (Liu et al., 2020; Lukić et al., 2022).

**FIGURE 2:**
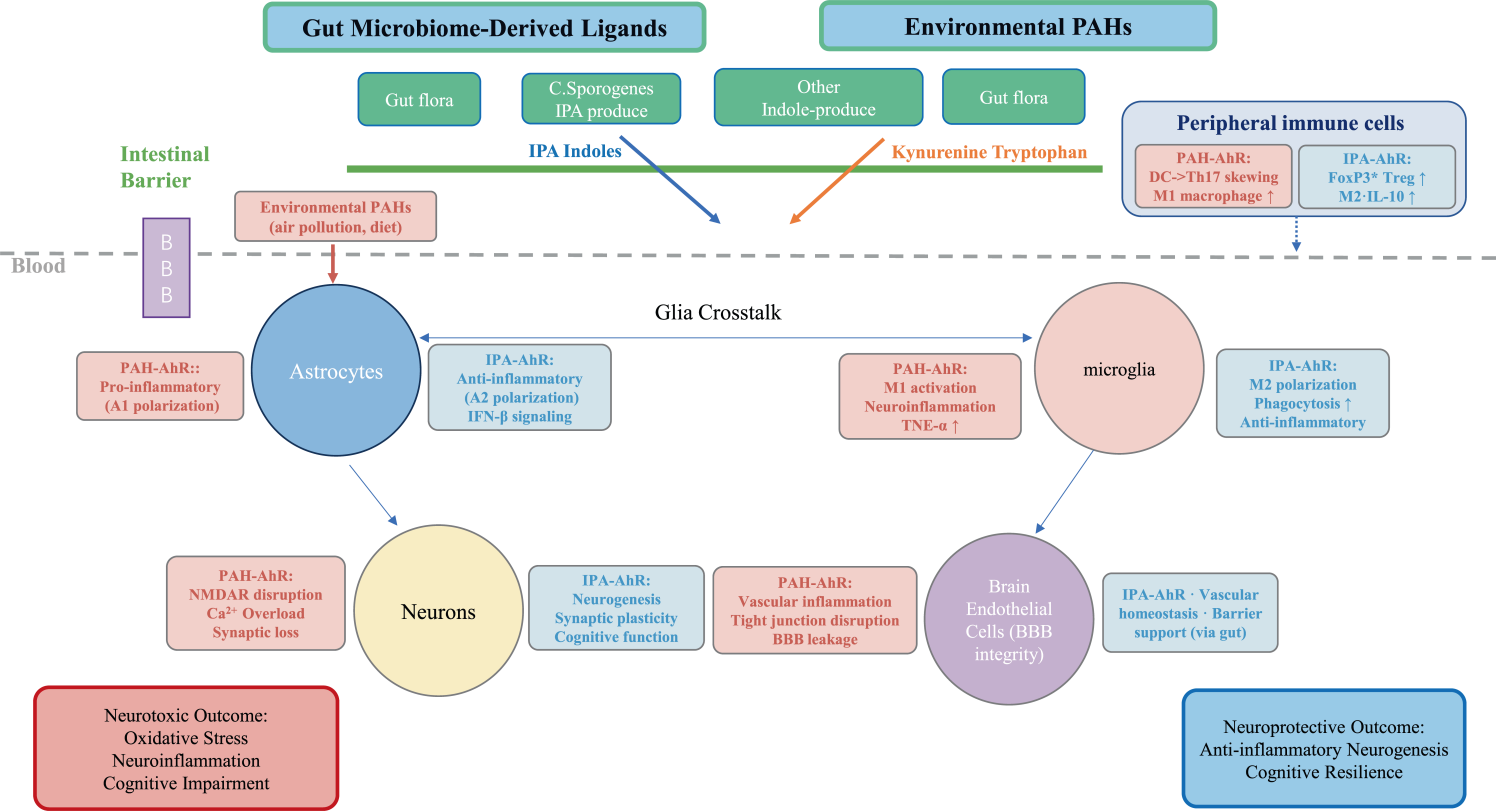
Cell type-specific actions of aryl hydrocarbon receptor (AhR) ligands in the central nervous system.

In summary, AhR acts as a pleiotropic regulator across cognitive and neurodegenerative diseases. Its role is determined by its ligands (Figure 2 and Supplementary Figure 2). PAHs consistently cast AhR in a villainous role, promoting pathways of toxicity, inflammation, and neuronal dysfunction. In contrast, many gut-derived tryptophan metabolites direct AhR toward protective roles. They activate programs for detoxification, anti-inflammation, repair, and balance. Disease often occurs when this balance shifts toward neurotoxicity. This shift can come from greater environmental agonist exposure. It can also come from lost protective metabolites or pathological changes in host metabolism. Understanding these ligand-specific effects is critical. It supports the development of precise diagnostic and therapeutic strategies.

## 6 Targeting the AhR and gut-brain axis for neuroprotection

The dual nature of AhR signaling and its central position in the gut-brain axis presents unique opportunities and challenges for therapeutic intervention. Therapeutic strategies fall into three categories. The first is direct pharmacological modulation of AhR. The second is indirect modulation via diet and microbiome engineering. The third is combined approaches.

Direct pharmacological targeting of AhR is an active area of research. In the context of neuroprotection, the goal is often to achieve a controlled, context-specific activation using selective AhR modulators (SAhRMs). This goal, however, faces substantial pharmacological hurdles. Laquinimod, a drug developed for multiple sclerosis, exerts its beneficial effects in EAE by activating AhR (Kaye et al., 2016). However, laquinimod’s development has been halted due to cardiovascular adverse events in Phase III trials. This safety signal suggests that even “selective” AhR modulation may produce off-target effects in peripheral tissues, particularly the cardiovascular system where AhR plays crucial homeostatic roles. Conversely, AhR antagonists are being explored, particularly in oncology to block tumor-promoting AhR signaling in the tumor microenvironment (Campesato et al., 2020). In neurological conditions, antagonist use might be considered in scenarios where chronic activation by environmental or pathological endogenous ligands (e.g., indoxyl sulfate in CKD) is driving harm (Salminen, 2023). The development of brain-penetrant SAhRMs with favorable safety profiles is a critical next step. Several pharmacological limitations remain unresolved. Because AhR is widely expressed across tissues, systemic modulation may produce unpredictable off-target effects. In addition, many AhR ligands show poor pharmacokinetic profiles, making adequate CNS penetration difficult to achieve. SAhRM development is further complicated by ligand-dependent biased agonism, as structurally distinct ligands can trigger different transcriptional responses even in the same cell type. Moreover, most brain-targeted delivery strategies are still at the preclinical stage. These issues collectively underscore the challenges of developing safe and selective AhR-based therapies for neurological disorders.

Dietary intervention is a foundational strategy. Diets high in tryptophan and specific fibers support a healthy microbiome. This microbiome produces beneficial indoles such as IPA (Konopelski and Mogilnicka, 2022; Li et al., 2024; Owe-Larsson et al., 2025). The Mediterranean diet, for instance, is associated with higher plasma IPA levels (Konopelski and Mogilnicka, 2022). Prebiotics (e.g., xylan) that selectively nourish IPA-producing bacteria like *C. sporogenes* can be employed (Li et al., 2024). Probiotic supplementation offers another therapeutic avenue. Specific strains can produce AhR ligands or modulate the microbial community to favor their production (Park and Gao, 2024). Synbiotic combinations (probiotics + prebiotics) have shown promise in preclinical AD models, improving cognition and pathology alongside increasing IPA levels (Li et al., 2024). Nevertheless, the translational barriers facing microbiome-based interventions are substantial. The gut microbiome exhibits profound inter-individual variability driven by host genetics, age, diet, and prior antibiotic exposure; a prebiotic regimen that reliably elevates IPA in one population may have little effect in another (Sonnenburg and Bäckhed, 2016). Furthermore, orally administered IPA itself shows limited systemic bioavailability and poor CNS penetrance, meaning that direct supplementation may not reproduce the sustained concentrations achievable via microbial synthesis (Owe-Larsson et al., 2025). Fecal microbiota transplantation is more invasive. It can fully reset the ligand-producing microbiome. It has proven effective in gastrointestinal disorders. Researchers are now testing it for neurological conditions. The rationale is clear. Restoring a healthy microbiome can restore a balanced set of beneficial AhR-activating metabolites (Park and Gao, 2024). Lifestyle interventions also show promise: intermittent fasting improves cognition in diabetic mice through a gut microbiome-mediated mechanism involving elevated IPA levels (Liu et al., 2020), and may additionally promote nerve regeneration via an IPA-dependent pathway (Serger et al., 2022).

Combination therapies are especially promising. They target multiple points in the gut-brain axis. A combination of a diet or probiotic regimen and an AhR-targeted drug could be designed. Such a combination might produce synergistic effects. Additionally, since AhR activation can be enhanced by the metabolic environment—such as butyrate acting as a histone deacetylase inhibitor to potentiate AhR-mediated gene transcription (Modoux et al., 2022)—combinations of metabolites (e.g., SCFAs and tryptophan metabolites) could be designed.

The therapeutic potential extends beyond neurodegenerative diseases to neurodevelopmental and psychiatric disorders. Prenatal PAH exposure links to cognitive deficits (Perera et al., 2006, 2009). Preventive public health measures to reduce air pollution are therefore extremely important. Tryptophan metabolism is implicated in conditions such as depression and ADHD (Li et al., 2022; Lukić et al., 2022). Interventions that correct microbial dysbiosis and metabolite imbalances may serve as valuable adjuncts to standard therapies.

Biomarker development and patient stratification represent another dimension of translational realism. The heterogeneity of AhR ligand environments across individuals means that AhR-targeted therapies are unlikely to benefit all patients equally; a precision medicine framework is therefore required. Candidate biomarkers fall into three categories. Potential biomarkers include indole metabolite profiles such as IPA, IAA, and indoxyl sulfate, metagenomic assessment of tryptophan-metabolizing gut bacteria, and PAH metabolites including 1-hydroxypyrene detected in blood or urine. However, static metabolite measurements poorly reflect dynamic AhR signaling flux in target tissues. Moreover, no validated surrogate of brain AhR activity exists for clinical trials (Dong et al., 2020). Emerging approaches such as AhR-responsive reporter systems in peripheral blood mononuclear cells may offer more dynamic readouts, though these tools require extensive clinical validation before deployment in neuroprotection trials. Patient stratification may focus on individuals with gut dysbiosis, high PAH exposure, or disrupted tryptophan metabolism, such as reduced plasma IPA levels and altered quinolinic acid/KYNA ratios. These features may indicate a relative deficiency of protective AhR ligands. The feasibility of such stratification in large-scale trials depends on standardizing metabolomics and microbiome profiling pipelines. This remains a methodological challenge that the field has not yet fully resolved (Sonnenburg and Bäckhed, 2016). Cost and technical complexity may also limit the implementation of large-scale metabolomic and microbiome profiling in routine clinical settings.

In conclusion, targeting AhR and the gut-brain axis represents a paradigm-shifting strategy for neuroprotection. Rather than a single “magic bullet,” the most effective strategy may involve a personalized combination of dietary modulation, microbiome engineering, and possibly selective receptor modulators. This integrated approach aims to alter the balance of AhR signaling. It seeks to shift activity away from the neurotoxic axis driven by pollutants and toward the neuroprotective axis sustained by healthy host-microbiome metabolism.

## 7 Conclusion and future perspectives

The aryl hydrocarbon receptor acts as a critical molecular interpreter. It translates chemical signals from the external environment and internal metabolic milieu into profound effects on brain function. This review has delineated the starkly contrasting roles of two major ligand classes: environmental polycyclic aromatic hydrocarbons and gut microbiota-derived tryptophan metabolites. PAHs activate AhR in a sustained and often dysregulated manner. This instigates a neurotoxic cascade involving oxidative stress, disrupted neurotransmission, neuroinflammation, and epigenetic alterations, ultimately impairing cognitive development and function (Chepelev et al., 2015; Crépeaux et al., 2012; Knecht et al., 2017a). IPA and related indole metabolites generally promote neuroprotective AhR signaling, whereas the effects of KYNA are more variable and concentration-dependent (Owe-Larsson et al., 2025; Serger et al., 2022; Wei et al., 2021). This contrast highlights the importance of metabolic context in shaping AhR-mediated outcomes.

The receptor integrates signals from the gut microbiome, diet, and environmental exposures. Its output determines whether the balance tips toward neurodegeneration or neuroprotection. Diseases such as Alzheimer’s and Parkinson’s may involve a “double hit.” Harmful AhR ligands increase. Protective ligands decrease. This combination pushes AhR signaling into a maladaptive state (Cortés Malagón et al., 2024; Iwaniak et al., 2024; Salminen, 2023).

Future research must navigate several key frontiers to translate this knowledge into clinical benefit. First, better understanding of ligand-receptor dynamics is needed. How do different ligands produce distinct AhR conformations and co-factor recruitment, leading to unique gene expression profiles? Advanced studies will provide critical insights. Structural biology and transcriptomics can compare PAH and tryptophan metabolite effects across neural cell types. Second, the role of non-canonical AhR signaling in the brain needs more investigation. Its functions as an E3 ubiquitin ligase and its interactions with other signaling pathways (e.g., NF-κB, hypoxia response) in neurons and glia are underexplored territories with therapeutic potential (Farooqi et al., 2023; Sondermann et al., 2023).

Third, the development of reliable biomarkers is crucial. Measuring levels of specific AhR ligands in blood, cerebrospinal fluid, or stool could enable the identification of individuals with “AhR signaling dysfunction” as a subtype of cognitive impairment (Cortés Malagón et al., 2024; Dong et al., 2020; Iwaniak et al., 2024). We caution, however, that “AhR signaling dysfunction” is a hypothetical construct. We currently lack standardized assays for AhR activation status *in vivo*, and static metabolite measurements may poorly reflect dynamic signaling flux. Fourth, clinical trials are urgently needed. These include trials of probiotics/synbiotics designed to boost IPA production in patients with mild cognitive impairment (Li et al., 2024; Owe-Larsson et al., 2025). Yet designing such trials presents methodological challenges. Should we target IPA levels as the primary endpoint, or cognitive function? How do we account for the placebo effects prevalent in gut-brain studies? As well as early-phase trials of safe, brain-penetrant selective AhR modulators. The safety requirements for chronic AhR modulation in elderly populations—who often have compromised hepatic and cardiovascular function—will be stringent.

Finally, public health initiatives to reduce exposure to neurotoxic PAHs remain a critical and evidence-based strategy for primary prevention of cognitive deficits (Perera et al., 2006, 2009). In parallel, promoting dietary patterns that support a diverse, AhR ligand-producing microbiome could serve as a population-level strategy for building cognitive reserve. However, we must avoid the “victim-blaming” implicit in lifestyle recommendations. Dietary interventions require access to fresh foods and nutritional education that are unevenly distributed across socioeconomic strata.

Two distinct journeys may converge on the same cellular receptor. One leads from environmental toxin to cognitive deficit, the other from dietary fiber to brain resilience. Deciphering the complex language of AhR signaling offers not only a deeper understanding of brain health and disease but also a roadmap for novel, mechanism-based interventions that harness the protective power of our internal microbial ecology to safeguard the mind.
