# Supplementary material for: Ligand-specific duality of aryl hydrocarbon receptor signaling in cognitive health: from environmental neurotoxicity to microbiome-mediated neuroprotection
Source: Front Neurosci. 2026 Jun 24;20:1823961. doi: 10.3389/fnins.2026.1823961 (PMC13342219; doi:10.3389/fnins.2026.1823961)
Supplement: Supplementary file 1 [file Data_Sheet_1.docx]

Supplementary Material

# Supplementary Figures

**Figure 1. Ligand-Specific Divergent Signaling of Aryl Hydrocarbon Receptor (AhR) in the Brain**

**Supplementary Figure 1.** Ligand-specific divergent signaling cascades of the aryl hydrocarbon receptor (AhR) in neuronal cells.AhR activation outcome is determined by four contextual variables: ligand affinity and pharmacokinetics, cell-type identity and epigenome, temporal dynamics, and tissue-specific co-factors. Environmental PAHs drive sustained, high-affinity AhR activation, triggering oxidative stress, neuroinflammation, synaptic dysfunction, and epigenetic remodeling, ultimately leading to neurodegeneration. In contrast, gut microbiota-derived tryptophan metabolites (IPA, indole, KYNA) activate AhR transiently, suppressing neuroinflammation, promoting neurogenesis, and reinforcing BBB integrity, yielding neuroprotection. This dichotomy is context-dependent rather than absolute. AhR, aryl hydrocarbon receptor; BBB, blood-brain barrier; IPA, indole-3-propionic acid; KYNA, kynurenic acid; PAH, polycyclic aromatic hydrocarbon.

**Figure 2. Cell Type-Specific Actions of AhR Ligands in the Central Nervous System**

**Supplementary Figure 2.** *Cell type-specific actions of AhR ligands within the neurovascular unit and gut-brain axis.* Schematic illustration of how PAHs and microbiota-derived tryptophan metabolites (e.g., IPA) differentially modulate AhR signaling across distinct CNS cell populations. (Top) Gut microbiota, particularly Clostridium sporogenes, metabolize tryptophan to produce IPA, which crosses the intestinal barrier and blood-brain barrier (BBB). Environmental PAHs enter circulation through inhalation or dietary intake. Before crossing the BBB, these ligands act on peripheral immune cells, modulating the immune tone that drives CNS inflammation. *(Middle)* In astrocytes, PAH-AhR drives pro-inflammatory A1 polarization, whereas IPA-AhR promotes anti-inflammatory A2 phenotype and IFN-β signaling. In microglia, PAH-AhR induces M1 activation and TNF-α release, while IPA-AhR facilitates M2 polarization and phagocytosis. In neurons, PAH-AhR disrupts NMDA receptor function and synaptic integrity, whereas IPA-AhR supports adult hippocampal neurogenesis and synaptic plasticity. In brain endothelial cells, PAH-AhR compromises BBB integrity via tight junction disruption, while IPA-AhR maintains vascular homeostasis through eNOS activation. These cell-specific effects collectively determine the balance between neurotoxicity and neuroprotection.
